# Specific Quality of Life Questionnaire Validation in Patients with Colorectal Cancer

**DOI:** 10.3390/diagnostics14222481

**Published:** 2024-11-07

**Authors:** Florin Mihăileanu, Cosmin Andrei Cismaru, Ariana Anamaria Cordoș, Răzvan Alexandru Ciocan, Stefan Chiorescu, Ioana Constantinescu, Bogdan Stancu, Caius Breazu, Horațiu Coman, Ioana Berindan Neagoe, Claudia Diana Gherman

**Affiliations:** 1 Department of Surgery—Surgery II, “Iuliu Hatieganu” University of Medicine and Pharmacy, 400006 Cluj-Napoca, Romania; florin.mihaileanu@umfcluj.ro (F.M.); chiorescu.stefan@umfcluj.ro (S.C.); ioanaconstantinescu2003@yahoo.com (I.C.); bstancu@umfcluj.ro (B.S.); 2 Research Center for Functional Genomics, Biomedicine and Translational Medicine, “Iuliu Hatieganu” University of Medicine and Pharmacy, 400347 Cluj-Napoca, Romania; ioana.neagoe@umfcluj.ro; 3 Department of Surgery—Practical Abilities, “Iuliu Hatieganu” University of Medicine and Pharmacy, 400337 Cluj-Napoca, Romania; razvan.ciocan@umfcluj.ro (R.A.C.); gherman.claudia@umfcluj.ro (C.D.G.); 4 Romanian Society of Medical Informatics, 300222 Timisoara, Romania; 5 Department of Surgery—Anaesthetics, “Iuliu Hatieganu” University of Medicine and Pharmacy, 400006 Cluj-Napoca, Romania; breazu.caius@umfcluj.ro; 6 Vascular Surgery Clinic, Cluj County Emergency Hospital, 400006 Cluj-Napoca, Romania; horatiucoman@gmail.com

**Keywords:** colorectal cancer, quality of life, risk factors, prognostic factors

## Abstract

(1) Background: The quality of life of cancer patients is not only important for their well-being, but it has great influence on the overall survival and response to therapy, considering the adherence to treatment and follow-up. (2) Methods: This research is a prospective study conducted over a period of 6 months involving patients admitted in the Department of Surgery II, Cluj County Emergency Clinical Hospital. The specific questionnaire designed by us for patients with colorectal cancer contains questions about the quality of life and symptoms such as weight loss, pain, constipation, and diarrhoea. (3) Results: Our prospective study included in the analysis 50 patients with colorectal cancer. The CR 29 questionnaire outlined scores below 30 for sore skin, urinary incontinence, dysuria, faecal incontinence, flatulence, discomfort from bowel movement, sexual dysfunction and hair loss. The CR 30 functioning scale depicted high scores for cognitive (100%, 95% CI [0.91–1]), physical (88%, 95% CI [0.75–0.95]), and functional (88%, 95% CI [0.39–0.68]) domains and low scores (<50) for emotional (98%, 95% CI [0.88–0.99]) and social (100%, 95% CI [0.91–1]) functions. (4) Conclusions: The quality of life of patients with colorectal cancer was influenced by socio-economic status, smoking, surgical procedure, and neoplastic pathology.

## 1. Introduction

Colorectal cancer is one of the most frequently diagnosed cancers in recent decades, with over 900,000 deaths annually. Lifestyle changes and high-risk habits (smoking, high-fat food intake, and obesity) are predicted to account for up to 5 million new cases by the year 2035 [1]. Treatment options (endoscopic, surgical, neoadjuvant radiotherapy, systemic treatment, and chemo- or immunotherapy) are not adverse effect-free. Morbidity and lifestyle changes can lead to psychological, physical, functional, and social deficiencies, all of which markedly influence the quality of life (QoL) of patients with colorectal cancer (CRC).

QoL is not only important for the well-being of the neoplastic patient, but it also profoundly influences survival and treatment response, having a great impact on adherence to therapy. Literature studies have investigated multiple factors involved in the assessment of QoL in CRC, suggesting that the initial symptoms, surgical procedures, and the number of comorbidities have a significant influence on QoL. Over the last two decades, the incidence of CRC has increased, while the mortality rate has dropped significantly, largely due to improved early diagnosis and new oncological therapies [2,3].

The 5-year survival of patients with CRC has improved in recent decades to 56% in Europe and 66% in the United States [4]. Moreover, the patient’s expectations of survival have increased in the last decade, reaching 93.2% 5 years after diagnosis. This leads to an increasing incidence of patients living with CRC, with an estimated global incidence of over 3 million people within 5 years of diagnosis [5].

QoL involves a multidimensional, dynamic, and subjective patient-centred concept, encompassing physical, functional, emotional, and social well-being. Therefore, QoL is an important aspect of assessing the complex impact of the disease on patients, their families, and the community they live in [6].

The critical limit of the quality of life resides in the fact that, being a subjective concept by definition, personally evaluated by the patient, it is difficult to quantify efficiently and objectively. To assess QoL, the use of questionnaires reported by the patient or investigator has become a standard practice. However, the lack of an instrument considered to be the “gold standard” is reflected in the wide range of options available, generic or disease-specific [7].

QoL in colorectal cancer patients is associated with several factors. For a simplified discussion, these factors were divided into five main categories: socio-demographic characteristics, health factors, factors dependent on neoplastic pathology and surgical procedures, lifestyle factors, and others [8].

The most frequently used quality of life questionnaires are generic (SF-36—“Short Form 36”, SF-12—“Short Form 12” and EQ-5D—“EuroQol-5D”), specific to neoplastic pathologies (FACT-G—“Functional Assessment of Cancer Therapy—General”; EORTC QLQ-C30—“European Organization for Research and Treatment of Cancer Core Quality of Life Questionnaire C30”; and QoL-CSV—“Quality of Life Patient/Cancer Survivor Version”) and specific to colorectal cancer (EORTC QLQ-CR29—“European Organization for Research and Treatment of Cancer Core Quality of Life Questionnaire Colorectal 29”; mCOH-QoL—abbreviated version for patients without stoma; and FACT-C—“Functional Assessment of Cancer Therapy-Colorectal cancer”) [9,10].

## 2. Materials and Methods

This research is a prospective study conducted over a period of 6 months, from January until June 2022, involving patients admitted in the Department of Surgery II, Cluj County Emergency Clinical Hospital.

Patients diagnosed with colorectal cancer who underwent a curative surgical intervention in the clinic were included in this study. Also, their medical history and complete records had to be available in order to be accepted in this study. The exclusion criteria were applied to patients who did not undergo a surgical intervention and patients with other concomitant neoplastic pathologies. All the patients signed the informed consent.

Data collection included general patient data, admission reasons, medical history, hereditary background, information about the workplace, living conditions, and addictions (alcohol, tobacco use, and drug usage). For the evaluation of the quality of life, 4 evaluation methods were used.

The non-specific method of assessing the quality of life of patients with colorectal cancer was the SF-36 questionnaire (appliable to any patient), and the pathologically specific ones are the EORTC_QLQ_C30 and EORTC_QOQ_CR29 questionnaires. The fourth questionnaire used was a newly designed, specific questionnaire to assess the quality of life of patients with colorectal cancer.

The SF-36 questionnaire is the short version of the Medical Outcome Study (36-Item Short Form Survey Instrument), which is used as a health indicator for the general population. The questionnaire is not focused on a specific disease, and it can be applied to patients with different pathologies. The questionnaire focuses on 8 parameters: physical functioning (PF), role physical (RP), body pain (BP), general health (GH), vitality (VT), social functioning (SF), role emotional (RE), and mental health (MH) [11].

The EORTC QLQ-C30 is the core questionnaire designed by the European Organization for Research and Treatment of Cancer, with 5 functional dimensions: physical functioning (PF), role functioning (RF), cognitive functioning (CF), emotional functioning (EF), and social functioning (SF). It also includes 3 symptoms scales (fatigue (FA), pain (PA), and nausea/vomiting (NV)), 6 single items addressing various symptoms, perceived financial impact, and a global health-related quality of life (HRQOL) subscale. Among the 30 items, the answers are on thought on a 7-point Likert scale [12].

The EORTC QLQ-CR29 was specifically designed by the EORTC QL Group (the European Organization for Research and Treatment of Cancer Quality of Life Group) as the QLQ-C30 supplement for the evaluation of HRQOL in colorectal cancer patients. This combination has already been widely used in both clinical and basic research [13,14]. The QLQ-CR29 includes 29 items that evaluate symptoms (gastrointestinal, urinary, pain, and others) and functional areas (sexual, body image, and others) that are associated with colorectal cancer and its treatments. There are separate items for patients with and without a stoma (items 49 to 54, with item 55 only for patients with a stoma) and items evaluating differentially the sexual function of men and women. The questionnaires make enquiries for all items regarding the past week, except those pertaining to sexuality, which request the patients to evaluate the items regarding the past four weeks. Similar to the EORTC QLQ-C30, the QLQ-CR29 has a Likert scale of four response categories (item 48 requires a ‘yes’ or ‘no’ answer) [10].

The specific questionnaire for assessing the quality of life of patients with colorectal pathology, which we designed, contains extra questions about signs and symptoms, such as weight loss, pain, constipation, and diarrhoea.

The statistical analysis was conducted based on the data type. Absolute and percent frequencies were used to summarise qualitative data. Quantitative data were summarised as mean-standard deviation for normally distributed data (Shapiro–Wilk test) and median (Q1–Q3) for non-normally distributed data, where Q1 = 25% percentile and Q3 = 25% percentile. For quantitative data, the Mann–Whitney test was used to compare groups, whereas chi-square tests were used for qualitative data. Using the Spearman correlation coefficient, the link between the items of the used quality of life questionnaire and the patient characteristics was evaluated. Statistical analysis was conducted using Statistica (version 8; StatSoft, Palo Alto, CA, USA) with a 5% significant level. *p* values less than 0.05 were statistically significant. Microsoft Excel was used to create graphical illustrations.

This study was approved by the Ethics Commission of the Cluj–Napoca County Emergency Clinical Hospital (approval No. 231/20.06.2022) and by the Ethics Commission of the University of Medicine and Pharmacy “Iuliu Hațieganu” Cluj–Napoca (approval No. 264/19.09.2022).

## 3. Results

The prospective analysis included 50 patients with colorectal cancer.

Within the investigated group, a higher percentage of patients were males (χ^2^ = 14.44, *p* < 0.001) (Table 1), with 60% of them coming from urban areas (60% [0.46–0.74]). Half of the investigated subjects had normal weight (χ^2^ = 19.86, *p* = 0.1) and were admitted for an elective intervention (χ^2^ = 94.74, *p* = 0.1). The age distribution of the selected group shows that the majority (66% 95% CI [0.52–0.78]) of them were older than 60 years of age, with a third of them being older than 70 years of age (32% 95% CI [0.2–0.46]). About two-thirds of the patients were non-smokers (62% [0.48–0.76]) and non-alcohol consumers (62% [0.48–0.76]).

The dominant (30%, 95% CI [0.18–0.45]) comorbidities were represented by heart disorders (χ^2^ = 7.22, *p* = 0.0072), as the rest of the pre-existent conditions are detailed in Table 2. The most frequent reasons for admission were pain (27 patients—54%), lower gastrointestinal bleeding (21 patients—42%), weight loss (20 patients—40%), and anaemia (19 patients—38%). T3 was the most frequent cancer stage at admission with 44% of the cases (95% CI [0.30–0.57]) (Table 3). For half of the patients included in this study, their tumour was localised on the sigmoid colon (χ^2^ = 0, *p* < 0.001). Over half of the patients (χ^2^ = 0.98, *p* = 0.322) had a colectomy, out of which the majority underwent a segmental colectomy (48%, 95% CI [0.30, 0.67]). Multiorgan resection was performed on seven patients (χ^2^ = 24.5, *p* < 0.001). A manual suture was performed for two-thirds of the patients (χ^2^ = 3.38, *p* = 0.066). Two patients presented postoperative complications (χ^2^ = 40.5, *p* < 0.001). Approximately 28% of the subjects have undergone neoadjuvant chemotherapy, 95% CI [0.16–0.42] (χ^2^ = 8.82, *p* = 0.003), while only three patients had neoadjuvant radiotherapy (χ^2^ = 36.98, *p* < 0.001). Oral feeding was resumed on postoperative day 2 for two-thirds of the patients (95% CI [0.46–0.74]), and 54% resumed transit on day 2 after surgery (95% CI [0.4–0.68]).

Quality of life questionnaire results.

**Figure 1:**
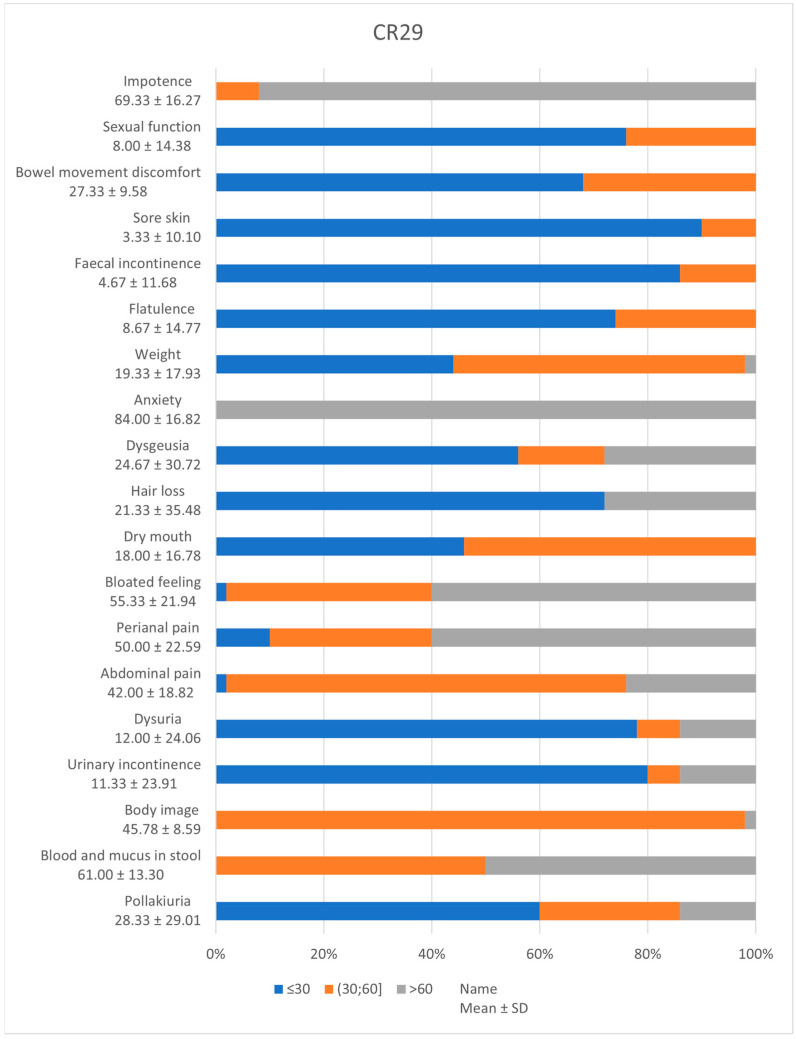
CR 29 incidence of symptoms.

**Figure 2:**
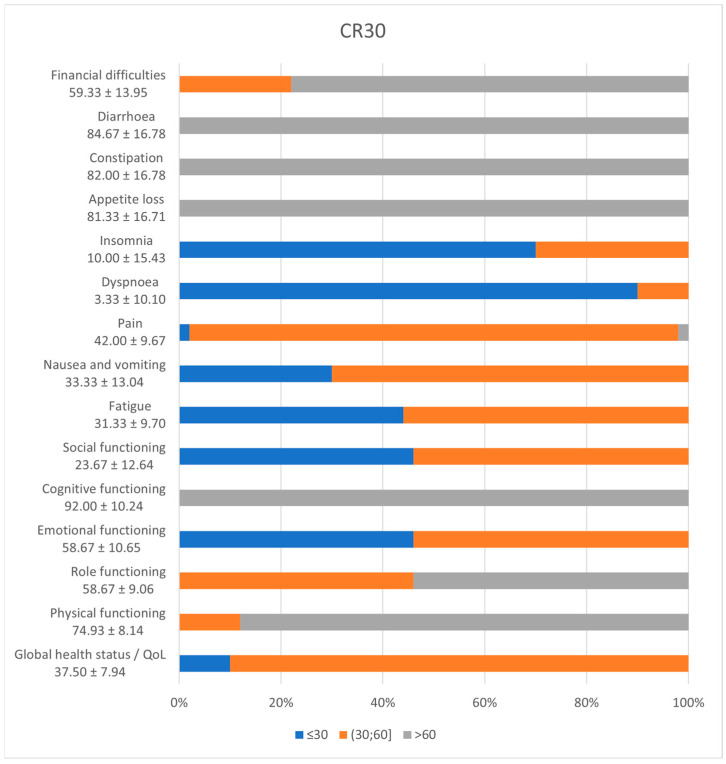
CR30 incidence of symptoms.

CR29 and CR30 questionnaires are specially designed for the assessment of the quality of life of patients with colorectal cancer. Figure 1 and Figure 2 depict the frequency of scores from the patient’s answers.

CR 29 outlined scores below 30 for sore skin (90%, 95% CI [0.77–0.96]), urinary incontinence (80%, 95% CI [0.66–0.90]), dysuria (78%, 95% CI [0.64–0.88]), pollakiuria (60%, 95% CI [0.45–0.73]), faecal incontinence (86%, 95% CI [0.73–0.94]), flatulence (74%, 95% CI [0.59–0.85]), discomfort due to bowel movement (68%, 95% CI [0.53–0.80]), sexual disfunction (76%, 95% CI [0.62–0.86]) and hair loss (72%, 95% CI [0.57–0.83]). High incidences of dysgeusia (56%, 95% CI [0.73–0.94]) and dry mouth (46%, 95% CI [0.32–0.61]) were encountered. At the opposite pole, almost all patients scored high for anxiety and 92% (95% CI [0.80–0.97]) impotence.

CR 30 functioning scales depict high scores for cognitive (100%, 95% CI [0.91–1]), physical (88%, 95% CI [0.75–0.95]), and role (88%, 95% CI [0.39–0.68]) functions and low scores (<50) for emotional (98%, 95% CI [0.88–0.99]) and social (100%, 95% CI [0.91–1]) functions. The global health status has a mean of 37.50 ± 7.94. The symptoms scale shows that all patients scored underneath 50 for fatigue, dyspnoea, insomnia, nausea, and vomiting, but above 60 for appetite loss, constipation, and diarrhoea and 78% (95% CI [0.64–0.88]) for financial difficulties.

**Figure 3:**
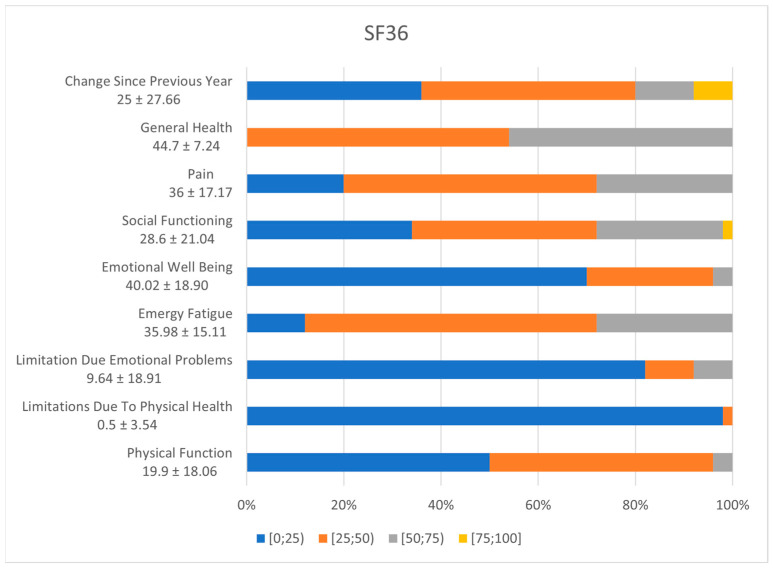
SF36 incidence of symptoms.

Evaluating the quality of life of patients with colorectal cancer, we analysed the results of the non-specific questionnaire SF36, based on nine parameters. The graphic in Figure 3 illustrates over 50% of the patients with low scores for physical functioning (50%, 95% CI [0.37–0.63]) and emotional wellbeing (70%, 95% CI [0.55–0.82]). Furthermore, almost all patients face limitations due to physical health (98%, 95% CI [0.88–0.99]) or emotional problems (82%, 95% CI [0.68–0.91]).

**Figure 4:**
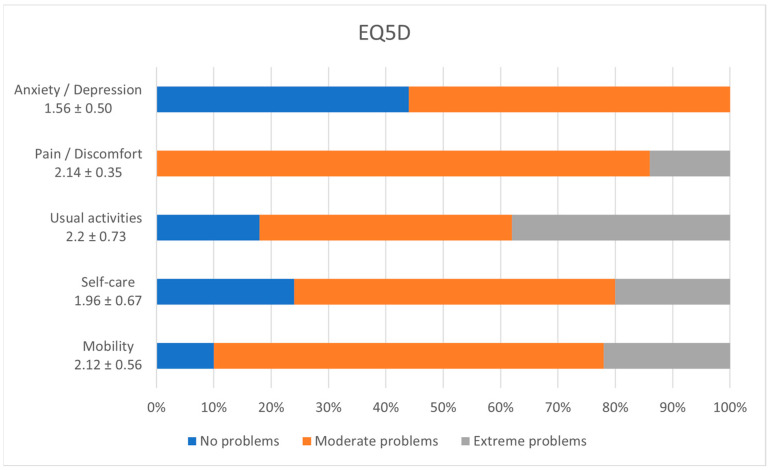
EQ5D incidence of symptoms.

Figure 4 details the resulting scoring of the EQ5D questionnaire, outlining that most of the patients in the group faced moderate problems with pain and discomfort (86%, 95% CI [0.73–0.94]), anxiety and depression (56%, 95% CI [0.73–0.94]), self-care (56%, 95% CI [0.41–0.70]), mobility (68%, 95% CI [0.53–0.80]), and usual activities (44%, 95% CI [0.30–0.59]).

## 4. Discussion

The present study included a number of 50 patients, 15 females and 35 males. A study conducted in 2014 in Spain shows that colorectal cancer is more common in male patients, affecting about 8.2% of men and 5.17% of women, with a recurrence of about 50% at 10 years after initial diagnosis. The positive predictive value (PPV) for cancer was 4.7%. The CRC detection rate was 2.75 per 1000 projections [15].

The age predictability of this type of cancer can be observed in our study, as out of the total number of 50 patients, 66% (95% CI [0.52–0.78]) were older than 60 years. A study conducted in 2018 showed that colorectal cancer occurs mainly between 68 and 72 years of age [16].

Regarding the urban vs. rural distribution, we found a number of 30 (60%, 95% CI [0.46–0.74]) patients from the urban area and a number of 20 (40% [0.26–0.54]) from the rural area. In a study conducted in 1992 on 678 patients, it was statistically demonstrated that the number of patients with colorectal cancer from rural areas is equal to that from urban areas [17].

Regarding the body mass index, in our study there were 27 normal-weight patients, 17 overweight, and 6 patients with grade I obesity (Table 1). According to a study conducted in 2013, it was outlined that an increased body mass index is a risk factor for colorectal cancer [18].

Toxics consumption was monitored, both alcohol and smoking. Two thirds (62%, 95% CI [0.48–0.76]) of the patients denied alcohol consumption. In a study conducted in 2019, it was shown that mild and moderate pre-diagnostic alcohol consumption was associated with better overall survival in colorectal cancer, and mild pre-diagnostic alcohol consumption has been associated with decreased colorectal cancer-specific mortality. Two-thirds (62%, 95% CI [0.48–0.76]) of patients in our group declared no tobacco use. A study conducted in 2018 reported that smoking is a risk factor in the development of colorectal cancer [4,19].

Symptoms were monitored (Tabel 1), and the main symptom reported by patients was pain, occurring in 27 patients. According to a 2021 article, pain is present in more than 70% of patients with advanced colorectal cancer [20]. Another frequent symptom was lower GI bleeding, where we identified a number of 21 patients who had lower digestive haemorrhage and 29 patients without any episode of macroscopic bleeding [21].

A research study from 2014 proposed chronic constipation as a potential risk factor for colorectal cancer development. A biological explanation for this connection is considered to be an increased contact of the carcinogens from the stool with the colonic mucosa. In our study, 26% (95% CI [0.14–0.4]) of patients confirmed constipation, while 8% (95% CI [0.02–0.19]) had diarrhoea [21].

Out of a total of 50 patients, 14 required chemotherapy treatment. According to a study conducted in 2014 on 114 patients, diarrhoea occurs more frequently in patients during chemotherapy for colorectal cancer than those without need of systemic treatment. The risk is higher during the first exposure, suggesting a variable susceptibility [21].

According to a 2022 study, weight loss may be a clinical sign of early-onset colorectal cancer, which also prevailed as a dominant admission symptom in our cohort of patients (Table 2) [22]. The sigmoid colon was the most common localisation for cancer in our group, followed by the inferior rectum and ascending colon.

Table 3 highlights that T3 tumour stage at hospital admission was the most prevailing tumour stage encountered in 44% of patients, 95% CI [0.30–0.57]. Due to this fact, colectomy with subsequent lymphadenectomy was performed for most of our patients (58%, 95% CI [0.44–0.72]), as shown in Table 2 [23].

The EQ-5D questionnaire developed in Europe is a widely used instrument for assessing general quality of life. The main result of our study shows that most of our patients are complaining of moderate anxiety, moderate pain, moderate to extreme problems regarding usual activities, and moderate issues with self-care and mobility [24,25].

Regarding the SF36 questionnaire, the lower the score, the greater the disability. In other words, a score of zero equals maximum disability, and a score of 100 equals no disability. Therefore, most of our patients grumble about changes in physical functioning, limitations due to physical health and due to emotional problems, and psychological well-being [26].

The CR29 questionnaire asked the subjects to list their symptoms that occurred in the previous week. Scores can be linearly transformed to give a score between 0 and 100. Higher scores indicate better functional status and a higher level of symptom control on the symptom scale. According to the patients’ answers, the most affected systems are the excretory, the reproductive, and the gastrointestinal. The patients are complaining of lower sexual function, faecal and urinary incontinence, pollakiuria, and discomfort due to increased bowel movements. Among these, we can also list blood and mucus in stool, pain (abdominal and perianal), and bloating as further drawbacks. The up-to-date literature also reveals that rectal (rather than colon) cancer has a negative impact on sexual health and gastrointestinal function but has no effect on overall QoL [27].

CR30 scores for all scales and single-item measures range from 0 to 100. A high scale score indicates a higher level of response [28]. Therefore, a high functional scale score represents a high/healthy level of functioning; a high global health status/QoL score represents a high QoL; but a high symptom scale/item score represents a high level of symptomatology/problems. From the study results, the cognitive and physical functions are not affected, but the social and emotional ones are. Moreover, the major problem for these patients is social reintegration. Looking at the symptoms, the most upsetting ones are insomnia, dyspnoea, pain, nausea, vomiting, and fatigue [29].

CRC has a very high prevalence worldwide; hence, economic assessment is gaining importance for evaluation of the cost-effectiveness of therapies in order to better advise resource allocation. There are a variety of metrics available for calculating health utility ratings. The influence of life happiness in this cohort may be improved by a higher degree of ERAS protocol compliance. The streamlined ERAS route at our institution may have contributed to the decrease in surgical stress, hence mitigating the influence of QOL on postoperative outcomes. Indeed, an intensively administered ERAS programme might counteract and conceal the impact of preoperative low QOL by providing individualised assistance [30].

In rectal cancer, emotional and social functioning were considerably impaired. The prevalence of symptoms was greater in rectal cancer. Nauseas, discomfort, insomnia, anorexia, constipation, and diarrhoea exhibited statistically significant differences. At inclusion, the global health status score in stage III was considerably lower. Only anorexia was more prominent among female patients. A German research study found no significant differences between colon and rectal cancer localisation regarding the health-related QOL, nor between male and female patients. An Austrian study focused on the effect of gender on quality of life and found no gender-specific illness response [31,32].

Several limitations of the present investigation should be mentioned. This is a heterogeneous sample cohort. One bias may be that patients with higher QoL and physically well-fitted were more likely to engage in this kind of trial. The QoL, anxiety, and depression were based on self-reporting, so, while these measures are frequently used in epidemiological and clinical research, it is impossible to remove the inherent biases associated with self-reporting. Also, this research was conducted on CRC survivors from Romania, which may restrict its applicability to other groups. Additionally, the research was constrained by the potential ceiling impact of physical activity intervention.

## 5. Conclusions

(a) QoL in colorectal cancer patients is determined by socio-demographic characteristics (patients from urban areas have a higher risk of developing colorectal cancer than people from rural areas).
(b) Health factors (an elevated BMI is a risk factor for the development of colorectal cancer).
(c) Neoplastic pathology and surgical procedure-related factors.
(d) Lifestyle factors (smoking may be a risk factor for colorectal oncogenesis; moderate to heavy alcohol consumption raises the chance of developing colorectal cancer; also, the level of stress, advanced age, and gender influence the quality of life of patients with colorectal cancer).

The identification of new, modern therapeutic solutions that can improve the therapeutic index of colorectal cancer patients could significantly improve their health-related QoL. Means to enhance treatment outcomes and methods to reduce mortality and morbidity from colorectal cancer could directly impact patients QoL and also have a social impact for the communities from a better reintegration of patients’ social involvement and employment.

## Figures and Tables

**Table 1:** General demographics.

	No. (%, [95% CI])
Gender	
Male	35 (70% [0.56–0.82])
Female	15 (30% [0.18–0.44])
Body Mass Index	
Normal Weight	27 (54% [0.4–0.68])
Overweight	17 (34% [0.22–0.48])
Obesity	6 (12% [0.04–0.24])
Admission type	
Elective	43 (86% [0.74–0.94])
Emergency	6 (12% [0.04–0.24])
Transfer	1 (2% [0–0.1])

**Table 2:** Hospitalisation and surgery-related data.

	No. (%, [95% CI])	χ^2^ (*p*)
Pathological history		
Previous neoplasia	1 (2% [0–0.11])	44.18 (*p* < 0.001)
Colonic polyposis	4 (8% [0.02–0.19])	33.62 (*p* < 0.001)
Colorectal diverticula	2 (4% [0.01–0.14])	40.5 (*p* < 0.001)
Diabetes mellitus	5 (10% [0.03–0.22])	30.42 (*p* < 0.001)
Gallstones	3 (6% [0.01–0.17])	36.98 (*p* < 0.001)
Gynaecological disorders	1 (2% [0–0.11])	44.18 (*p* < 0.001)
Heart disorders	15 (30% [0.18–0.45])	7.22 (*p* = 0.007)
Metabolic and endocrinological disorders	2 (4% [0.01–0.14])	40.5 (*p* < 0.001)
No associated conditions	9 (18% [0.09–0.31])	19.22 (*p* < 0.001)
Previous surgeries	2 (4% [0.01–0.14])	40.5 (*p* < 0.001)
Renal disorders	4 (8% [0.02–0.19])	33.62 (*p* < 0.001)
Reason for admission		
Anaemia	19 (38% [0.25–0.53])	2.42 (*p* = 0.119)
Constipation	13 (26% [0.14–0.4])	10.58 (*p* < 0.001)
Constipation-diarrhoea alternance	11 (22% [0.12–0.36])	14.58 (*p* < 0.001)
Diarrhoea	4 (8% [0.02–0.19])	33.62 (*p* < 0.001)
Inapparent (inappetence?)	12 (24% [0.13–0.38])	12.5 (*p* < 0.001)
Lower gastrointestinal bleeding	21 (42% [0.28–0.57])	0.98 (*p* = 0.322)
Pain	27 (54% [0.4–0.68])	0.18 (*p* = 0.6714)
Stoma	2 (4% [0.01–0.14])	40.5 (*p* < 0.001)
Sub-occlusion syndrome	2 (4% [0.01–0.14])	40.5 (*p* < 0.001)
Tenesmus	3 (6% [0.01–0.17])	36.98 (*p* < 0.001)
Tumour	11 (22% [0.12–0.36])	14.58 (*p* < 0.001)
Weight loss	20 (40% [0.26–0.55])	1.62 (*p* = 0.203)
Tumour location		
Ascending colon	9 (18% [0.09–0.31])	19.22 (*p* < 0.001)
Descending colon	1 (2% [0–0.11])	44.18 (*p* < 0.001)
Sigmoid colon	25 (50% [0.36–0.65])	0 (*p* < 0.001)
Superior/middle rectum	3 (6% [0.01–0.17])	36.98 (*p* < 0.001)
Inferior rectum	11 (22% [0.12–0.36])	14.58 (*p* < 0.001)
Frozen pelvis/carcinomatosis	1 (2% [0–0.11])	44.18 (*p* < 0.001)
Surgical intervention		
Colectomy	29 (58% [0.44–0.72])	0.98 (*p* = 0.322)
Anterior rectal resection	15 (30% [0.18–0.45])	7.22 (*p* = 0.007)
Colostomy	4 (8% [0.02–0.19])	33.62 (*p* < 0.001)
Abdomino-perineal rectal resection	2 (4% [0.01–0.14])	40.5 (*p* < 0.001)
Anastomosis		
Manual	32 (64% [0.5–0.78])	3.38 (*p* = 0.066)
Mechanical	12 (24% [0.12–0.38])	12.5 (*p* < 0.001)
No	6 (12% [0.04–0.24])	27.38 (*p* < 0.001)

**Table 3:** Tumour staging.

T Stage	M0 Stage	M1 Stage	Total
T1	2 (4% [0–0.14])	0 (0% [0–0.08])	2 (4% [0.01–0.14])
T2	10 (20% [0.1–0.34])	0 (0% [0–0.08])	10 (20% [0.1–0.34])
T3	17 (34% [0.22–0.48])	5 (10% [0.04–0.22])	22 (44% [0.30–0.57])
T4	7 (14% [0.06–0.26])	9 (18% [0.08–0.32])	16 (32% [0.2–0.46])

## Data Availability

The data generated in the present study may be requested from the corresponding author.
